# Forcing Ahead: Second-Line Treatment Options for Lenalidomide-Refractory Multiple Myeloma

**DOI:** 10.3390/cancers17071168

**Published:** 2025-03-30

**Authors:** Katia Mancuso, Simona Barbato, Francesco Di Raimondo, Francesca Gay, Pellegrino Musto, Massimo Offidani, Maria Teresa Petrucci, Elena Zamagni, Renato Zambello, Michele Cavo

**Affiliations:** 1 IRCCS Azienda Ospedaliero-Universitaria di Bologna, Istituto di Ematologia “Seràgnoli”, 40138 Bologna, Italy; katia.mancuso3@unibo.it (K.M.); simona.barbato3@unibo.it (S.B.); e.zamagni@unibo.it (E.Z.); 2 Dipartimento di Scienze Mediche e Chirurgiche, Università di Bologna, 40138 Bologna, Italy; 3 Divisione di Ematologia, Azienda Ospedaliero-Universitaria Policlinico di Catania, Scuola di Specializzazione in Ematologia dell’università di Catania, 95125 Catania, Italy; diraimon@unict.it; 4 Divisione di Ematologia 1, AOU Città Della Salute e Della Scienza di Torino, 10126 Torino, Italy; francesca.gay@unito.it; 5 Dipartimento di Biotecnologie Molecolari e Scienze per la Salute, Università degli Studi di Torino, 10126 Torino, Italy; 6 Unit of Hematology and Stem Cell Transplantation, Department of Precision and Regenerative Medicine and Ionian Area, “Aldo Moro” University School of Medicine, AOUC Policlinico, 70124 Bari, Italy; pellegrino.musto@uniba.it; 7 Clinica di Ematologia, Unità di Trapianto di Cellule Staminali e Terapia Cellulare dell’AOU delle Marche, 60126 Ancona, Italy; massimo.offidani@ospedaliriuniti.marche.it; 8 Ematologia, Azienda Ospedaliera Universitaria Policlinico Umberto I, 00161 Roma, Italy; petrucci@bce.uniroma1.it; 9 Unità di Ematologia, Dipartimento di Medicina (DIMED), Università di Padova, 65100 Padova, Italy; r.zambello@unipd.it

**Keywords:** multiple myeloma, relapsed/refractory multiple myeloma, lenalidomide refractoriness, MM therapy, second-line treatment in MM

## Abstract

The therapeutic landscape for multiple myeloma has gradually expanded in recent decades, leading to unprecedented deep and sustained responses as well as remarkable improvements in patient survival. Nonetheless, changes in treatment algorithms have raised new demands for patients with relapsed/refractory disease, as prior exposure and refractoriness to prior therapies impact the choice of subsequent treatments. In particular, refractoriness to lenalidomide—an established backbone of treatment in both front-line and maintenance settings and a key component of many approved regimens used in relapsed disease—is associated with suboptimal clinical outcomes. Therefore, identifying the most appropriate management in lenalidomide-refractory patients, and even more so in patients who are refractory to more than one agent, is critical. At present, treatment options for this growing subgroup of patients are still limited; however, recent data from clinical research are promising. Herein, we summarized the currently available treatment options and discuss future directions based on the latest results from ongoing clinical trials.

## Simple Summary

The inclusion of novel agents in the therapeutic armamentarium of multiple myeloma over the last decade has revolutionized the history of the disease, leading to unprecedent deep and sustained responses, ultimately translating into remarkable improvements in patient survival. Nonetheless, the management of patients with relapsed/refractory disease remains critical. In particular, one of the most challenging areas in the evolving treatment landscape for MM is identifying the most appropriate management for patients who are refractory to lenalidomide, a growing population of individuals with suboptimal clinical outcomes. To address this issue, we herein summarized the treatment options currently available for these patients, and discuss the future directions based on latest results from ongoing clinical trials.

## 1. Introduction

The inclusion of new drug classes in the therapeutic armamentarium of multiple myeloma (MM) has led to remarkable improvements in patient survival. Nonetheless, the natural history of the disease continues to be marked by sequential phases of remission and relapses, which are driven by increasing drug refractoriness at each subsequent line of therapy (LoT) and leave MM as an uncurable disease for most patients.

To overcome these barriers, clinical research has explored innovative treatment strategies with new mechanisms of action (MoAs), the most promising being T-cell redirecting therapies, which showed substantial efficacy in heavily pretreated patients with relapsed/refractory (RR) disease. In this context, however, it is important to emphasize that the management of MM is becoming increasingly complex as the number of treatment options expands. Indeed, prior exposure and resistance to one or more of novel drug class(es) impact the choice of the subsequent LoTs.

In particular, one of the most challenging areas in the evolving treatment landscape for MM is identifying the most appropriate management for patients who are refractory to lenalidomide (len), a growing population of individuals with suboptimal clinical outcomes. To more carefully address this issue, we herein summarize the treatment options currently available in this setting and discuss the future directions based on the latest results from ongoing clinical trials.

## 2. Refractoriness to Lenalidomide in MM: A Growing Problem to Face the Disease

The therapeutic landscape in MM has gradually expanded over the last two decades due to the progressive availability of three classes of novel agents, including the proteasome inhibitors (PIs) bortezomib, carfilzomib, and ixazomib; immunomodulatory agents (IMiDs) thalidomide, lenalidomide, and pomalidomide; and monoclonal antibodies (MoAbs) targeting CD38 (daratumumab and isatuximab) and SLAMF7 (elotuzumab) antigens. These novel drugs have been combined with each other to form highly effective three-drug (triplet) or four-drug (quadruplet) regimens that have become the mainstays of therapy for both newly diagnosed (ND) and RRMM. In the meantime, early exposure to one or more classes of novel agents has brought up new challenges related to the choice of subsequent treatment option(s) for those patients who are drug(s) refractory at the time of relapse. In particular, the widespread use of continuous lenalidomide as part of front-line therapy for transplant-eligible (TE) and -ineligible (TI) patients has progressively increased the probability of refractoriness to this agent at the time of first relapse, highlighting the need for effective second-line salvage regimens.

According to the International Myeloma Workshop Consensus Panel 1, refractoriness to len is defined as less than partial response or progressive disease either during therapy or within 60 days of its discontinuation [1,2]. Noteworthy, when these definitions were proposed, many treatment regimens actually used in the real world, including triplets and quadruplets, were not available. In addition, maintenance or continuous therapy were not a standard-of-care approach. As of today, most NDMM patients, either TE or TI, are continuously exposed to len at different doses, in the range between 10 and 25 mg/daily, as part of upfront therapy, and the majority of them are len refractory at the time of their first relapse. Therefore, resistance to len currently is a problem that needs to be faced when choosing a treatment plan.

Whether patients relapsing while treated with low doses (e.g., 5–15 mg once daily) of len can be considered refractory to the drug compared to those progressing on full dose (i.e., 25 mg, once a day) has been debated for a long time. There are no definitive data supporting that a possible increase in the dose of len can overcome resistance to the drug. Several retrospective analyses have shown the lack of any relationship between clinical outcomes and the dose of len at the time of relapse. In particular, relapse while on lower doses (5–15 mg) was associated with poor prognosis, and results from len retreatment at first relapse were similar in patients who were refractory to standard or lower doses [3,4]. In a study aimed at gaining insights into the different patterns of len resistance, a longer exposure (≥1 year) to len and longer interval (≥18 months) from last len dose to subsequent start of pomalidomide were associated with higher response rates and longer PFS and OS, highlighting the importance of optimal sequencing to face the challenge of IMiD refractoriness [5].

Moreover, concerns have been raised about the possibility of len retreatment after a wash-out period in patients who discontinued treatment due to adverse events (AEs). In a recent study, it was found that the probability of len discontinuation within the first year of treatment was approximately 33%, most frequently due to fatigue and cytopenias. Interestingly, in a subset of these patients, retreatment with len at the time of subsequent relapse was successful in approximately 50% of cases, suggesting that patients and physicians are willing to more easily accept toxicities at the time of relapse [6]. Nevertheless, in some cases, it may be necessary to move toward len-sparing treatment regimens–similar to the treatment strategies adopted for len-refractory patients–to avoid the risk of recurrence of AEs.

Overall, the increasing frequency of refractoriness to len in first-line settings–and, to a lesser extent, the occurrence of toxicity associated with len-based therapies–collides with the limited availability of len-free regimens in subsequent LoTs. Recent data on treatment outcomes in len-refractory RRMM with ≥1 previous LoT provided further evidence that both clinical trials and real-world outcomes are suboptimal in these patients, compared with the outcomes achieved in those who are len sensitive [7]. Indeed, data from clinical trials showed that progression-free survival (PFS) and overall survival (OS) were shorter in the len-refractory cohort (months: 8.8 [n = 2699] and 21.7 [n = 1066], respectively) than the intent-to-treat (ITT) population (months: 13.8 [n = 5380] and 35.9 [n = 2264], respectively). In particular, median PFS (mPFS) in the len-refractory cohort with one prior LoT ranged between 11.9 months (n = 12) and 19.1 months (n = 111). Moreover, despite considerable variation, real-world data collectively indicate that patients who were refractory to len had worse outcomes, especially the most pretreated ones and those who were refractory to multiple drug classes, underscoring an unmet clinical need in real-world clinical practice and the need for new approvals.

## 3. Mechanisms of Resistance to Lenalidomide

Len is a second-generation IMiD with more potent anti-MM activity and less relevant neurological effects than its precursor thalidomide. The main target of IMiDs is a ubiquitin ligase complex called CRL4^CRBN^ which is formed of Cullin-4 (CUL4), RING box protein ROC1, DNA damage-binding protein1 (DDB1), and cereblon (CRBN) proteins. The latter acts as the substrate complex for CRL4^CRBN^ and, once structurally rearranged by the binding of IMiDs to its surface, triggers the ubiquitination and subsequent degradation of B cell transcription factors Ikaros (IKZF1) and Aiolos (IKZF3). This ultimately leads to downregulation of interferon regulatory factor 4 (IRF4) and c-MYC, which are critical for myeloma cell survival [8,9,10,11,12,13,14,15]. Acquired CRBN mutations have been reported in at least one-third of len–and pomalidomide–refractory patients, thus representing the single most relevant contributor to the emergence of drug refractoriness. These mutations may result in the partial or complete loss of CRBN function, which may be, at least in part, rescued by administering the novel CRBN binding agents (CELMoDs) mezigdomide and iberdomide. Studies applying in vitro models suggested the possible role of epigenetic changes in the development of CRBN downregulation and len resistance, which was also found to be associated with tetraploidy and the loss of one additional copy of the 3p region containing the CRBN locus [16,17,18,19,20,21,22]. Interestingly, non-coding RNAs (micro-RNAs or circRNAs) have also been described to be involved with diminished drug sensitivity or resistance, likely by activating or downregulating upstream or downstream signaling cascades. Notably, the expression of certain clusters of differentiation (CD), like CD138 or CD44, or of other surface antigens, including the glucose-regulated protein (GRP) 78, were found to be associated with len resistance [23,24,25]. Last, but not least, direct or indirect modulation of other pathways were identified as possible contributors to the development of resistance. Among these, the upregulation of cyclin-dependent kinase 6 (CDK6), the reduced expression of nuclear receptor co-repressors 2 (NCOR2), the upregulation of the MEK/ERK pathway by modulating the tumor necrosis factor receptor-associated factor 2 (TRAF2), or interleukin 6 (IL-6)-mediated activation of the JAK/STAT3 pathway were identified as major factors that may modulate sensitivity or resistance to len in MM in either in vitro or in vivo studies [14,26,27]. Hopefully, further study in the field will certainly shed light on the main mechanisms underlying len resistance and, more importantly, how to overcome or prevent this barrier in the future.

## 4. Treatment Strategies Against Recurrence: Current Weapons in Len-Refractory MM

Treatments at first relapse are aimed at achieving the deepest level and a longer durability of responses as a way to prolong PFS and OS. The choice of salvage therapy at the time of relapse is primarily driven by resistance to prior drug(s); however, other factors are also important. In addition to patient-related factors (the most important being age, frailty status, and presence of comorbidities), a careful evaluation of the characteristics of relapse, organ function, depth and duration of response to previous regimen(s), along with all due considerations on the onset—or persistence—of any toxicity related to previous treatments might be of help in selecting the optimal drug sequencing. Then, considerations regarding regulatory aspects, efficacy, and feasibility of therapy should be made.

Since the choice of first-line therapy dictates, at least in part, the selection of second-line therapy, the EHA-ESMO recommendations and IMWG guidelines published in 2021 suggested considering different options for len-refractory RRMM, according to the partner(s) of len as part of the upfront therapy. Since len-based triplets including either bortezomib or daratumumab were mostly used at that time, possible clinical scenarios at the time of first relapse in both the TE and TI settings were basically represented by refractoriness to len as single agent or dual refractoriness to len combined with either bortezomib or daratumumab [28,29]. Overall, currently licensed len-sparing regimens include a PI or an IMiD in their backbones, as summarized in Table 1.

### 4.1. PI-Based Doublets and Triplets Incorporating Either an Anti-CD38 MoAb or an Inhibitor of Exportin 1

Following the first head-to-head study showing the superiority of carfilzomib and dexamethasone (Kd) over bortezomib and dexamethasone (Vd), subsequent randomized studies confirmed that the addition of an anti-CD38 monoclonal antibody like daratumumab (D) or isatuximab (isa) to Vd or Kd, respectively, significantly improved clinical outcomes compared to the standard-of-care doublet comparators. However, patients refractory to len, and even more so those progressing on len frontline, were underrepresented in these studies, and the superior PFS afforded by the triplets regimens was often not statistically significant, suggesting the need for further analyses of numerically adequate subsets of these patients.

In the ENDEAVOR study, 929 patients with 1–3 previous LoTs were randomized to receive either Kd, with K administered at 56 mg/m^2^ (Kd56), or Vd. Results showed a mPFS of 18.7 months with Kd56 versus 9.4 months with Vd; ORR was 77% versus 63%, respectively [30,31,32]. Results on 235 patients (25% of the total population) with len-refractory disease were subsequently reported and failed to show statistically significant differences between the two groups in terms of PFS (median 8.6 vs. 6.6 months) and OS (median 29.2 vs. 21.4 months). However, subgroups analyses indicated reduced risk of death with Kd56 in the 12–34% range across all subgroups, including patient age, cytogenetic risk, previous len exposure, and len-refractory status.

In the CASTOR trial, the addition of D to Vd (DVd) significantly increased the median (m) PFS (16.7 months) compared to Vd (7.1 months), especially when used as second-line therapy (27.0 vs. 7.9 months, respectively), and MRD negativity rates (14% vs. 2%) [33,34,35]. However, in the subgroup of 60 patients refractory to len, regardless of prior LoTs (representing 24% of the total population), mPFS with DVd was clearly suboptimal (7.8 months compared to 4.9 months in the Vd group). Thrombocytopenia (46.1% vs. 32.9%), neutropenia (13.6% vs. 4.6%), anemia (16.0% vs. 16.0%), and pneumonia (10.7% vs. 10.1%) were the most common grade 3–4 treatment emergent adverse events (TEAEs), while infections occurred in 29.6% and 19.0% of patients in the DVd and Vd arms, respectively. However, the rate of discontinuation due to TEAEs or infections remained low and similar between groups (TEAEs: 10.7% vs. 9.3%; infections: 2.9% vs. 2.1% in the two groups, respectively). Although DVd was a widely used regimen in the past, particularly before the subsequent availability of more active triplets, data on len-refractory patients at first relapse are scarce. To help fill that gap, an Italian retrospective real-life study evaluated the efficacy and safety of a second-line DVd regimen in 85 len-refractory patients [64]. With a median follow-up of 25 months, the median PFS and OS were 15 and 47 months, respectively; the overall response rate (ORR) was 86%, including 61% with a very good partial response (≥VGPR) or better. In line with other findings, the dose of prior len did not affect PFS.

The Kd combination was further evaluated in combination with the anti-CD38 MoAbs daratumumab and isatuximab in the phase 3 registrative CANDOR (DKd vs. Kd) and IKEMA (isaKd vs. Kd) trials for RRMM patients with 1–3 prior LoTs.

In the CANDOR study, the addition of D to Kd improved mPFS (28.4 vs. 15.2 months) and overall QoL compared to Kd. Notably, data upon extended follow-up to >4 years reinforce the favorable risk-benefit profile of the DKd combination in terms of PFS, OS improvement trend (Hazard Ratio, HR, 0.78), and MRD status, consistent with previous reported results [36,37,38]. Interestingly, pre-planned and post-hoc subgroup analyses confirmed a PFS benefit and a trend toward longer OS in len-refractory patients (32% of the total population), with mPFS values in those after 1 and ≥2 prior LoTs of 25 vs. 11.1 months and 28.1 vs. 12 months, respectively, and mOS not reached with DKd vs. 38.2 months with Kd. Regarding the toxicity profile, grade ≥3 thrombocytopenia (24.7% vs. 16.3%), anemia (17.5% vs. 16.3%), and upper respiratory tract infections (3.9% vs. 1.3%) were likely to be moderately higher with the triplet regimen. However, treatment discontinuations were similar in the two groups (34% of patients in the DKd and 27% in the Kd arm discontinued any study treatment because of AEs).

Despite the small sample size and the lack of comparison arms, results from PLEIADES and EQUULEUS studies investigating on the efficacy of the DKd combination further supported DKd as a standard treatment regimen for RRMM, including len-refractory disease [65].

Results from the IKEMA study, with an extended median follow-up of 44 months, showed a mPFS of 35.7 months, 87% ORR, including 44.1% complete response (CR), and 33.5% MRD negativity in the isa-Kd group. In the control group, mPFS was 19.2 months, CR was 28.5%, and MRD negativity was 15.4%. No difference between treatment arms could be appreciated in terms of OS [39,40,41,42,43,44]. Of note, a recent subgroup analysis confirmed a consistent benefit with isa-Kd over Kd in terms of PFS (HR, 0.59), CR (38.6% vs. 11.9%), and MRD negativity (24.6% vs. 9.5%) rates also in len-refractory patients (representing 32% of the enrolled population). Regarding safety, more patients in the isa-containing group experienced grade ≥3 neutropenia (19% vs. 7%), pneumonia (21% vs. 14%), and upper respiratory tract infections (40% vs. 28%), especially in aged patients, but treatment discontinuation was similar in the two groups (14% and 18% in the isa and control groups, respectively) [44]. Recently published results from a real-world study of 103 RRMM patients after 1–3 prior LoTs who received isa-Kd outside clinical trials were consistent with those of the IKEMA trial [66]. As expected, due to the evolving treatment landscape in MM, len-refractory patients were more represented in the real-life study than in the IKEMA trial. In particular, among patients who received len, 20 (19%) were len-exposed and 73 (71%) len refractory, reinforcing the robustness of isa-Kd in a real-world setting.

Regarding more recent available therapies, selinexor (S), the first-in-class orally inhibitor of exportin 1, in combination with Vd (SVd) was approved by the US Food and Drug Administration (FDA) and the European Agency of Medicine (EMA) for adults with RRMM who have received ≥1 prior therapy. Indeed, initial results of the BOSTON phase 3 trial demonstrated significant improvements in mPFS and ORR with SVd vs. Vd in patients with previously treated MM [45], and this superiority was retained across all efficacy endpoints and various subgroups, including frail, elderly, and high-risk patients. With an extended follow-up of 28 months, twice as long as initially reported, mPFS was 13.2 months for SVd and 9.5 months for Vd, while the respective mOS values were 36.7 vs. 32.8 months. In the same post-hoc analysis, the clinical outcomes of 106 len-refractory patients (representing 26% of the total population) were also evaluated. Results confirmed the superiority of SVd over Vd in terms of mPFS (10.2 vs. 7.1 months), mOS (26.7 vs. 18.6 months), and ORR (67.9% vs. 47.2, including ≥VGPR rates of 35.8% vs. 24.5%), highlighting the importance of a double mode-of-action switch [46]. Based on these results, the 2021 EHA-ESMO MM guidelines recommend SVd as a second-line option both for len-sensitive and len-refractory patients and for those previously treated with daratumumab (independently of sensitivity to len). As for toxicity, TEAEs across all grades were consistent with the BOSTON primary analysis and often required S dose modifications (89% of cases), need for supportive therapy for associated toxicities, or treatment discontinuation (median time to discontinuation in len-refractory patients: 6.1 vs. 4.7 months for SVd and Vd arms, respectively). Notable, the once-weekly dosing schedule of V in the SVd arm was associated with significantly lower rate and severity of peripheral neuropathy compared with those observed with the twice-weekly administration of V in the control arm [45,67]. Being that SVd approval was quite recent, real-world data with this therapy are limited. However, data from a recent real-world analysis on AEs, predominantly including gastrointestinal disorders, symptoms of myelosuppression, and various non-specific manifestations, alert physicians to the need to further investigate the safety profile of selinexor [68].

Patients with t(11;14) or high BCL-2 expression may benefit from venetoclax-containing therapies. In this sense, the phase 3 BELLINI study compared venetoclax (Ven) combined with bortezomib and dexamethasone (Ven-Vd) with Vd in RRMM with one median prior LoT, including 20% of patients who were len refractory [69]. At a median follow-up of 45.6 months, mPFS in the ITT population was significantly longer in the Ven-Vd combination compared to controls (23.4 vs. 11.4 months, respectively), especially in patients with t(11;14) (36.8 vs. 9.3 months). However, no statistical significance could be appreciated in the len-refractory cohort. The median OS was not reached in either arm among all patients. However, potentially due to increased toxicity and risk of infections, OS was inferior in the Ven-Vd arm, except for patients with t(11;14), again emphasizing the role of venetoclax in this subset of patients [70]. However, the subsequent phase III CANOVA trial, which evaluated Ven plus dexamethasone (Ven-d) with respect to pomalidomide plus dexamethasone (Pd) in patients with t(11;14)-positive RRMM failed to show a benefit in terms of PFS, the primary endpoint of the study. Indeed, at a median follow-up of approximately 2 years, Ven-d numerically prolonged PFS by more than 4 months (mPFS 9.9 vs. 5.8 months in the two arms respectively), but the difference was not statistically significant, leaving an uncertain future for Ven in the MM therapeutic landscape, in spite of the biological rationale [71].

The elotuzumab (elo)-Vd combination was tested in an open-label phase 2 study in which this association was compared to Vd, but the results were disappointing [72].

### 4.2. IMiD-Based Triplets Incorporating an Anti-CD38 or -SLAMF7 MoAb or a PI

Regarding second-line combinations with the third-generation IMiD pomalidomide, the association of pomalidomide (P) with Vd (PVd) poses an alternative. Indeed, in the phase 3 OPTIMISMM trial, including 559 patients who had received 1–2 prior LoTs, with 226 receiving 1 prior LoT (among these, 57.1% were refractory to len). Up to 70% of all patients were len refractory, and PFS and ORR were significantly improved with PVd versus Vd (mPFS: 11.7 vs. 6.9 months; ORR: 82% vs. 50%) [47,48,49]. Interestingly, second-line PVd significantly improved PFS in len-refractory patients with a mPFS of 17.8 months compared with 9.5 months in the Vd arm. Moreover, 56.3% of len-refractory patients achieved ≥VGPR in the PVd (compared with 23.1% in the Vd arm), further supporting the use of PVd regimen as a treatment option for MM patients at first relapse, including those who had progressed after frontline len. In this subgroup of patients, grade 3–4 TEAEs occurred in 90.6% (PVd) and 71.0% (Vd) of patients, cautioning against potential toxicity when selecting this treatment option. Specifically, neutropenia (35.9% vs. 12.9%), thrombocytopenia (17.2% vs. 22.6%), peripheral sensory neuropathy (9.4% vs. 3.2%), and infections (29.7% vs. 21.0%) were the most common grade 3–4 hematologic and nonhematologic TEAEs (PVd vs. Vd, respectively).

Alternatively, DPd is another regimen that has been shown to be effective at first relapse in patients with len-refractory disease. Results from the phase 3 randomized APOLLO trial showed that DPd reduced the risk of disease progression or death compared with Pd alone in patients who had received at least one previous LoT (mPFS: 12.4 vs. 6.9 months, respectively; ORR: 69% vs. 46%) [50,51]. Interestingly, about 80% (n = 120) of the 151 patients enrolled in this study were len refractory, and subgroup analyses showed that mPFS superiority with the triple versus double regimen was retained in these patients (9.9 vs. 6.5 months). In addition, improvements in pain, functional status, and disease-related symptoms were reported, underscoring the role of combinations of daratumumab plus pomalidomide in this context [52].

DPd was also investigated in the MM-014 phase 2 trial, with 112 patients after 1 to 2 prior treatment lines, 75% of whom (n = 84) were len refractory, mainly (70 out of 112) relapsed after frontline len. The mPFS was 23.7 months in the whole population receiving DPd and 23.0 months in len-refractory patients; however, mPFS in patients relapsing after 1 LoT was not specified [53,54]. Notably, these data also align with real-world data showing a mPFS of 18.9 months in patients progressing on len and receiving second-line DPd [73].

The combinations of Pd with MoAbs targeting CD38 and SLAMF7, like isa or elo (i.e., isa-Pd and elo-Pd, respectively), may be considered in heavily pretreated patients. Such combinations were approved by the EMA in the context of RRMM patients who have failed ≥2 previous LoTs (3 previous median lines), by inclusion criteria c necessarily exposed to bortezomib and len, based on the results of the two studies ICARIA and ELOQUENT-3.

Specifically, 307 patients who had received at least two previous LoTs including len and a PI were randomized to receive isa-Pd (n = 154) or Pd (n = 153) in the ICARIA trial [55]. Notably, almost all patients (94%) were refractory to len at study entry, and 77% were refractory to a PI. With a median follow-up of 35.3 months, mPFS was longer in the isa-Pd than in the control group (11.1 vs. 5.9 months), and a higher ORR and depth of response were also reported with the triplet regimen [56]. Overall, TEAEs occurred in both groups (74% and 61% of patients, respectively). Although treatment discontinuation was infrequent, dose reductions were more frequent in the isa-Pd arm versus control group (76% vs. 47% for pomalidomide and 68% vs. 51% for dexamethasone); pneumonia was the most frequent serious AE (all grades, both groups: 23% for isa-Pd and 21% in controls) [56,57,58]. Of note, with a longer follow-up of approximately 52 months, no new safety concerns were identified, and the 6.9-month improvement in mOS afforded by isa-Pd (24.6 months vs. 17.7 months with Pd) reinforced the clinical benefit of this triplet regimen for patients refractory to len and a PI.

Lastly, current options also contemplate the combination of Pd with elo as a third-line option, based on the results achieved in the phase 2 ELOQUENT-3 trial, enrolling 117 RRM patients with ≥2 prior LoTs, 90% of whom were refractory to len. In the trial, the elo-Pd groups showed superiority over Pd in terms of mPFS, ORR, and patients achieving at least a VGPR (10.3 vs. 4.7 months, 53% vs. 26% and 20% vs. 9%, respectively). In addition, at 45 months of follow-up, elo-Pd was superior in terms of OS than Pd (29.8 vs. 17.4 months) [59]. Among AEs, the most common grade 3–4 were anemia (11.7% vs. 21.8%) and neutropenia (15.0% vs. 27.3%), with serious AEs occurring in 70.0% and 60.0% of patients (elo-Pd vs. Pd group, respectively), including respiratory tract infection (8.3% vs. 5.5%) and pneumonia (6.7% vs. 9.1%). However, a recent real-world study on the efficacy of elo-Pd in a large cohort of 247 patients facing the major therapeutic challenge of dual refractoriness to daratumumab and lenalidomide reported modest efficacy outcomes in terms of PFS (6.6 months) and OS (17 months). However, these results were likely to be adversely influenced by the unfavorable prognostic features that characterized by chance this series of patients [74,75].

Overall, these trials showcased the potential of pomalidomide-based combinations in the setting of len-refractory patients, though latter combinations refer to patients who were more refractory to treatment than those included in several of the previously mentioned studies. Certainly, double-refractory patients have fewer chances, but len-free combinations with an alternative anti-CD38 and carfilzomib or pomalidomide may be advised in these cases. However, when patients acquire resistance to len, PI and anti-CD38 agents (i.e., triple-class refractory, TCR) in the early stages, the issue is amplified, as this scenario has not yet been sufficiently investigated in recent trials, and no standard therapy has been defined thus far. Importantly, the MAMMOTH study reported an OS of 8.6 months in TCR patients, paralleled by the mOS of 12.4 months reported in the LOCOMMOTION study in TCR (73.8% of total) patients, highlighting the urge to find new solutions soon.

Given the lack of evidence, treatment choice is guided by multiple factors, including prior therapies, patient status, presence of comorbidities, and drug availability, at the physician’s discretion. Kd-based regimens may be an option, provided that no cardiovascular risk factors are present; otherwise, the aforementioned PVd, SVd, and elo-Pd (and, for those with t(11;14), Ven-Vd) may be alternatives. However, results in terms of PFS are still unsatisfactory, and new strategies should therefore be individualized and approved as soon as possible. Other possible scenarios that should caution in treatment selection include extra-medullary disease in patients presenting with plasmacytomas or plasma cell leukemia, those with high-risk cytogenetics, and patients carrying renal impairment, for whom additional factors to the above must be considered. Moreover, available data from clinical trials remain limited and not always consistent. Indeed, while the treatment of len-refractory patients remains challenging, the management of these difficult-to-treat patients poses an even greater unmet clinical need. In this regard, the variability of high-risk definitions across trials, the scarcity of studies specifically dedicated to high-risk MM patients, and the small size of high-risk subgroups in broader trials make it difficult to establish strong, evidence-based therapeutic recommendations. As a result, a risk-adapted treatment approach remains an unattainablegoal. Likely, the integration of innovative immunotherapies, such as CAR-T cell therapies, BsAbs, and antibody–drug conjugates (ADCs), into earlier lines of treatment could likely help improve the poor prognosis of these patients, but further studies in these settings are awaited.

## 5. T-Cell Redirecting Therapies and Immunoconjugate (Belantamab Mafodotin)-Based Triplets Reshaping the Future

T-cell redirecting therapies, including CAR T-cell and BsAbs, directly harness immune cells to target novel proteins expressed on the surface of plasma cells, the most relevant of these being actually B-cell maturation antigen (BCMA), G protein–coupled receptor class C group 5 member D (GPRC5D), and Fc receptor-like protein 5 (FcRH5). Even before the ADC belantamab mafodotin (bela), these breakthrough therapies with new MoAs were initially tested in stage heavily pretreated triple-class- and penta-refractory RRMM and subsequently moved in earlier phases of the disease for the management of RRMM after one or two prior LoTs (Table 2). Phase 2 and 3 studies specifically designed to explore their use upfront after the diagnosis of MM have been established, are ongoing, or are planned, and preliminary results from these trials are eagerly awaited.

Regarding BCMA-targeting CAR-T cell therapies, a single infusion of idecabtagene vicleucel (ide-cel; Abecma^®^) was superior over standard therapies (i.e., DPd, DVd, ixazomib-Rd, Kd or elo-Pd, at physician’s discretion) in the phase 3 KarMMa-3 study, enrolling patients with a median of 3 (range 2 to 4) prior LoTs and triple-class refractoriness in 66% of them. PFS, the primary study endpoint, was significantly longer in the ide-cel group (mPFS: 13.3 vs. 4.4 months), and these patients had a higher chance of ≥CR (44% vs. 6%) and ≥CR plus MRD-negativity (35% vs. 2%, at 10^−5^ sensitivity) than the control group. The mOS was 41.4 months in the ide-cel arm and 37.9 months in the standard care group. Overall, a manageable toxicity profile was reported for the ide-cel-treated group [72]. At final PFS analysis, with a median follow-up of 30.9 months, ide-cel retained the PFS benefit previously seen in comparison with standard regimens (13.8 vs. 4.4 months), and the best outcome was seen in patients with 2 prior LoTs (16.2 vs. 4.8 months, respectively) [73]. Based on these results, EMA granted expanded approval to include the treatment of triple-class-exposed (TCE) RRMM patients who have received at least 2 prior therapies, representing the first CAR T-cell therapy to gain approval in earlier lines of therapy in the European Union in this setting. In addition, ide-cel has demonstrated a favorable risk-to-benefit ratio in earlier stages of the disease with frequent, profound, and sustained responses in patients with NDMM progressing within 18 months after treatment initiation, in the KarMMa-2 cohorts 2a and 2c, with a manageable safety profile. However, final data are awaited [82,83].

In parallel, in the phase III CARTITUDE-4 trial, a single infusion of ciltacabtagene autoleucel (cilta-cel, Carvykti) significantly prolonged PFS and OS over standard therapies (i.e., PVd or DPd at physician’s choice) in double-exposed (i.e., exposed to PIs and IMiDs) len-refractory patients after 1–3 previous LoTs. At a median follow-up of 33.6 months, mPFS and mOS were not reached. The 30-month probability of PFS was 59.4% in the cilta-cel arm and 25.7% in the control group, while respective estimates of OS were 76.4% vs. 63.8% [60,61]. Overall, a 70% reduction in the risk of disease progression or death favoring the cilta-cel arm compared with the standard regimens was seen in the broad patient population. This benefit was retained also in len-refractory patients, who had a HR of 0.55 for PFS. More patients in the cilta-cel arm compared to the control group achieved ≥CR (73.1% vs. 21.8%) and MRD negativity (60.6% vs. 15.6%). Deeper responses and a more durable MRD negative status afforded by CAR T-cell therapy ultimately improved the QoL of treated patients, with the advantage of a considerable treatment-free-interval beyond improved outcomes. As a result, the EU Commission has approved an extension of the indication of cilta-cel for the treatment of adult patients with RRMM who have received at least one prior therapy, including an IMiD and PI, who have demonstrated disease progression with the last therapy and are len refractory. Moreover, in April 2024, cilta-cel was approved as second-line treatment in the United States for len-refractory patients after at least one prior LoT, including a PI and an IMiD [84].

Meanwhile, BsAbs against BCMA, GPRC5D, andFcRH5 are emerging as highly effective treatments in len-refractory patients after one or more LoTs; however, results from ongoing investigational studies have yet to be published. At the same time, the anti-BCMA ADC belantamab mafodotin (B), initially tested as monotherapy in the DREAMM-2 trial [85] and subsequently compared with Pd in the DREAMM-3 study [86], was further evaluated in the large phase 3 randomized trials DREAMM-7 and DREAMM-8 in the setting of len-exposed and len-refractory RRMM patients after at least one prior LoT.

In the DREAMM-7 study, 494 patients with progression of MM after ≥1 prior LoT(s) (53% with prior exposure to len and 34% len-refractory) were andomized to receive BVd (n = 243) or DVd (n = 251). After a median follow-up of 39.4 months, BVd demonstrated a significantly longer mPFS vs. standard-of-care DVd (36.6 vs. 13.4 months, respectively) [76]. In addition to PFS, the primary study endpoint, BVd compared to DVd conferred improvements in the secondary efficacy endpoints, including ≥CR (35% vs. 17%), CR plus MRD negativity (25% vs. 10%), duration of response (DOR: 35.6 vs. 17.8 months), and PFS2 (the HR was 0.56 [0.41–0.76]) [78]. The clinical benefits afforded by BVd were retained across many subgroups, including len-refractory patients who had a mPFS of 25 months (vs. 8.6 months with DVd), 35% CR (vs. 11% with DVd), and 42% MRD negativity (vs. 13% with DVd) [78]. Notably, at the second interim analysis, mOS was not yet reached in both arms, and 36-month estimations of OS were 74% in the BVd group and 60% in the DVd group, suggesting an early and statistically significant benefit with BVd (the HR was 0.58 [95% CI, 0.43–0.79]) [79]. Regarding safety, grade ≥ 3 AEs occurred almost in all (95%) patients with BVd, consistent with previously reported data, and were serious in half. In particular, ocular side effects, which occurred in 79% of bela-patients, were effectively managed with dose modifications, including delays and reductions, and events of worsening visual acuity were mostly resolved. Results from the DREAMM-7 study support the potential future use of BVD at first relapse or later [79], pending its approval by regulatory agencies.

Similarly, in the phase 3, randomized, DREAMM-8 study bela-Pd (BPd) was associated with a significantly greater PFS benefit, as well as depth and durability of responses than PVd. Overall, 302 patients with RRMM after ≥1 prior LoT(s) were enrolled into the study. Of these, 155 (of whom, 53% and 35% after 1 and 2–3 prior LoT(s); 100% and 81% len-exposed and len-refractory, respectively) were randomized to receive BPd [80]. At a median follow-up of 21.8 months, mPFS was not reached in the bela-group (vs. 12.7 months in the PVd group), and the 12-month estimated PFS was 71% with BPd and 51% with PVd (the HR was 0.52, *p* < 0.001). ORR was 77% (40% ≥CR, 24% MRD negative) vs. 72% (16% ≥CR, 5% MRD negative), while follow-up for OS is ongoing (12-mos OS: 83% vs. 76%). Of note, among patients treated in the second line (n = 82 in the bela-arm, n = 77 in the PVd arm), mPFS was not reached with BPd and was 18.5 months with PVd (12-month PFS rate: 78% vs. 64%). The following additional endpoints were noted in these subgroups: ≥CR: 46% vs. 23% and MRD negativity 27% vs. 4%. Specifically, 119 len-refractory patients received second-line treatment (with either BPd [n = 66] or PVd [n = 53]). The main results from their comparisons for primary and secondary endpoints were as follows: mPFS not reached with BPd vs. 13.1 months with PVd (12-month PFS rates: 74% vs. 53%); 44% vs. 21% ≥CR; MRD negativity of 22% vs. 3%; and median DOR: not reached vs. 13.8 months [81]. Regarding toxicity, ocular events were common and occurred in 89% of the patients who received BPd (grade 3 or 4 in 43%). However, these were effectively managed by protocol-recommended dose modification of bela and were characterized by high rates of resolution, low treatment discontinuation rates, and the absence of a negative effect on patient-assessed QoL, thereby positioning bela-containing triplets to address the need for new regimens for the treatment of patients with myeloma at first relapse [80,81,87].

## 6. Discussion

The current treatment scenario in NDMM includes the widespread use of combination regimens that incorporate the three main classes of novel agents into triplet and quadruplet therapies for both TE and TI patients who are fit to tolerate them. Although these highly effective regimens have dramatically prolonged PFS and OS, most patients continue to relapse after prolonged exposure to one or more of these agents. The chance of being triple-class exposed since the first LoT actually involves an increasing number of NDMM patients, and most of them are likely to receive the second LoT at the time they have already become refractory to lenalidomide or to both lenalidomide and an anti-CD38 MoAb. The emerging issue of double-class refractoriness is out of the scope of this review and will not be extensively discussed. The finding of len refractoriness highlights the urgent need to address the barriers these patients continue to face by identifying viable treatments in a setting with still limited options [88].

PI-based triplets incorporating Vd or Kd combined with either daratumumab (DVd, DKd), isatuximab (isa-Kd), or selinexor (SVd), as well as regimens including pomalidomide and dexamethasone as a backbone to which either daratumumab (DPd) or bortezomib (PVd) and CAR T- cell therapy are added with ide-cel basically represent the main therapeutic strategies for len-refractory patients after the first LoT. A careful balance between the efficacy and toxicities of each of these options is crucial for the selection of the optimal treatment and sequencing and ultimately affects the physician’s choice.

The numerically longer mPFS values observed with anti-CD38 MoAbs combined with Kd in comparison with the other triplets might support their preferential use in patients who are len refractory and daratumumab naïve. In this regard, it is important to underscore that in the updated analysis of the IKEMA trial reporting an overall mPFS of 36 months, only the hazard ratio for PFS in the 32% of len-refractory patients was provided. This HR value overlapped with that of the whole patient population, a finding that might reinforce the preferential choice of this regimen for len-refractory patients. However, a limitation to the use of Kd-based triplets is represented by the frequent hospital admissions (i.e., twice weekly for 3 weeks in four-week cycles) to receive the intensive dosing schedule of intravenous (i.v.) carfilzomib, with added bi-monthly i.v. isatuximab from cycle 2 onwards, a barrier potentially mitigated by the once monthly administration of s.c. daratumumab in the DKd regimen from cycle 7 onwards. In addition, the approximately 3-fold higher probability of any grade and grade 3–4 cardiac failure seen with isa-Kd in the subgroup of patients ≥ 70 years compared to those aged less than 70 years raises the issue of the need to carefully select, or to exclude, older patients at risk of developing cardiovascular adverse events and eventually to follow them closely. Indeed, older and unfit patients, particularly those with pre-existing or concomitant cardiovascular disease, might be better candidates for Pd- or Vd-based regimens like PVd and SVd. Regarding both of these treatments, the twice-weekly dosing schedule of bortezomib (for two consecutive weeks over the first eight 3-week cycles) followed by a bi-weekly/cycle administration from cycle 9 onwards) in the PVd regimen poses again the problem of frequent hospital admissions, which might be partially overcome by the once-weekly administration of bortezomib in every 5-week cycle of the SVd regimen. In the OPTIMISMM and BOSTON studies, the rate of grade 3–4 peripheral sensory neuropathy was 8% with PVd and 5% with SVd; however, PVd is likely to be at higher risk of worsening a pre-existing neurological toxicity. DPd is safer and easier to use due to the advantage offered by the subcutaneous administration of daratumumab every 2 weeks from the third to the sixth four-week cycle and once monthly thereafter. However, refractoriness to len and pomalidomide as part of the first and second LoTs, respectively, limits the choice of a viable treatment option after the second relapse.

In double-exposed and len-refractory patients after 1–3 prior LoTs, a single infusion of cilta-cel induced high rates of deep (i.e., MRD negative) and durable remissions, ultimately prolonging PFS and OS compared to standard-of-care therapies and improving the QoL of treated patients due to the considerable treatment-free interval beyond improved outcomes. In view of these premises, cilta-cel has emerged as the most promising therapeutic option at first relapse and will undoubtedly be an important player in the near future. However, challenges regarding the actual feasibility of a CAR T-cell therapy persist, especially in a second-line setting, involving progressively larger numbers of patients. First and foremost, the limited number of centers qualified for CAR-T administration, along with the number of available aphaeretic slots (due to the workloads impacting both individual centers and cell factories) and dedicated hospital beds in each center, represent some of the major barriers for this breakthrough therapy. Also, the need for an integrated multidisciplinary approach and the management of short–-and long-term toxicities and complications potentially hamper their widespread practicability, especially in view of a predictably (very) high demand. Therefore, any approved CAR T-cell therapy for the care of patients after their first relapse will necessarily warrants consideration of these logistical and organizational challenges to provide for a review of organizational and structural planning by health policies.

Off-the-shelf products, like BsAbs, will represent an attractive and alternative option to the use of CAR T-cell therapies in earlier LoTs, provided that positive results will be released from ongoing clinical studies and that the optimal sequencing of T-cell redirecting therapies will be definitely established. Also, still pending issues related to the optimal management and mitigation strategies of toxicities related to the continuous administration of these products should be considered. Moreover, solid data are still lacking to establish with certainty the right sequencing to maximize efficacy results, and their use in early phases has yet to be assessed. In this regard, more data on the use of BsAbs in patients relapsed after anti-BCMA CAR-T cell therapy are currently available, as CAR-T therapies preceded the introduction of BsAbs, showing good efficacy in this context. By contrast, data on the use of CAR-T after BsAbs are scarce. Overall, there is sufficient evidence to advise against anti-BCMA CAR-T cell therapy in patients progressing with BsAbs directed against the same target. However, emerging data on the use of BsAbs as bridging therapy before CAR-T cell infusion are biologically and clinically compelling, but further data are needed in these settings. In view of these considerations and of promising early results from DREAMM-7 and DREAMM-8 studies, triplet-based belantamab mafodotin anti-BCMA therapies are likely to become potential competitors of T-cell redirecting therapies once licensed. In particular, both of these studies provided evidence for two highly effective options to be used in the second-line setting, offering considerable advantages in terms of drug availability, non-need for step-up dosing or hospitalization for their administration, and flexible scheduling that may be personalized according to individual necessities or potential side-effects.

Moreover, BVd or BPd may represent a viable alternative to cilta-cel in non-fit patients or to BsAbs after appropriate benefit/risk considerations in the infection risk. Treatment-related ocular adverse events, initially challenging although reversible, are now more easily manageable with regular monitoring, the possible support of an ophthalmologist, and appropriate dose delays or reduction. Meanwhile, other agents may also become available in the near future, and specific disease and patient characteristics will be critical in choosing the most appropriate therapeutic algorithm. Importantly, the potential need for sequential exposure to different therapies—the efficacies of which may be mutually influenced—as well as potential mechanisms underlying resistance, along with logistical challenges should be considered, with the ultimate goal of maximizing treatment efficacy

## 7. Conclusions

Overall, current treatment strategies have undoubtedly ameliorated the management of MM over time. Nonetheless, resistance to therapy, with particular regard to len refractoriness as early as in the first-line setting, has become a relevant issue in recent years, making it increasingly difficult to identify the optimal treatment algorithm in the context of RRMM patients, especially at first relapse. In this review, we focused on actual strategies in use for MM patients progressing under, or after, len-based regimens. Certainly, more data focusing on len-refractory population are needed. Meanwhile, based on promising results from recent studies, the approval of novel combinations is eagerly awaited, to augment the actual therapeutic armamentarium against MM, with the goal of making the idea of healing more and more possible in the future.

## Figures and Tables

**Table 1:** Currently licensed treatment strategies in len-refractory MM: data on clinical efficacy.

PI-Based Regimens														
Overall									Len-Refractory					
**Study** **[Ref]**	**Regimens**	**Prior LOTs** **(m)**	**mFU** **(mos)**	**mPFS** **(mos)**	**mOS** **(mos)**	**ORR** **(%)**	**≥CR** **(%)**	**MRD-** **(%)**	**Len-Ref** **(%)**	**mPF** **S** **(mos)**	**mOS** **(mos)**	**ORR** **(%)**	**≥CR** **(%)**	**MRD-** **(%)**
ENDEAVOR [30,31,32]	Kd56 vs. Vd	2 (1–3)	44.3	18.7 vs. 9.4	47.8 vs. 38.8 (51.3 vs. 43.7 as 2nd LOT)	77 vs. 63	13 vs. 6	/	25	8.6 vs. 6.6	29.2 vs. 21.4	/	15.5 (Kd56)	/
CASTOR [33,34,35]	DVd vs. Vd	2 (1–9)	72.6	16.7 vs. 7.1 (27.0 vs. 7.9 as 2nd LOT)	49.6 vs. 38.5	84 vs. 63	29 vs. 10	14 vs. 2	24	7.8 vs. 4.9	/	/	/	/
CANDOR [36,37,38]	DKd vs. Kd	2 (1–5)	50.6	28.4 vs. 15.2	50.8 vs. 43.6	84 vs. 73	22 vs. 8	28 vs. 9	32 vs. 36	28.1 vs. 11.1	NR vs. 38.2	79.8 (DKd)	/	/
IKEMA [39,40,41,42,43,44]	isaKd vs. Kd	2 (1–4)	56.6	35.7 vs. 19.2	NR vs. 50.6	87 vs. 84	44 vs. 29	34 vs. 15	32 vs. 34	HR 0.60 in favor of isaKd	/	/	39 vs. 12	25 vs. 10
BOSTON [45,46]	SVd vs. Vd	2 (1–3)	28	13.2 vs. 9.5 (21.0 vs. 10.7 as 2nd LOT)	36.7 vs. 32.8 (NR vs. 32.8 as 2nd LOT)	76 vs. 62	17 vs. 11	/	37 vs. 39	10.2 vs. 7.1	26.7 vs. 18.6	67.9 vs. 47.2	/	/
**IMiD** **-based regimens**														
**Overall**									**Len-Refractory**					
**Study** **[Ref]**	**Regimens**	**Prior LOTs** **(m)**	**mFU** **(mos)**	**mPFS** **(mos)**	**mOS** **(mos)**	**ORR** **(%)**	**≥CR** **(%)**	**MRD-** **(%)**	**Len-ref** **(%)**	**mPFS** **(mos)**	**mOS** **(mos)**	**ORR** **(%)**	**≥CR** **(%)**	**MRD-** **(%)**
OPTIMISMM [47,48,49]	PVd vs. Vd	2 (1–3)	64.5	11.7 vs. 6.9 (20.73 vs. 11.63 as 2nd LOT)	35.6 vs. 31.6	82 vs. 50	13 vs. 3	/	71 vs. 69	17.8 vs. 9.5 (2nd LOT)	29.8 vs. 24.2	85.9 vs. 50.8	/	/
APOLLO [50,51,52]	DPd vs. Pd	2 (1–5)	39.6	12.4 vs. 6.9	34.4 vs. 23.7	69 vs. 46	25 vs. 4	9 vs. 2	63 vs. 73	9.9 vs. 6.5	/	/	/	/
MM-014 [53,54]	DPd	2 (1–2)	41.9	23.7	56.7	78.6	26.8	/	76	23.0	53.6	77.6	22.4	/
ICARIA [55,56,57,58]	isaPd vs. Pd	3 (2–4)	52.4	11.5 vs. 6.5	24.6 vs. 17.7	60 vs. 35	9 vs. 2	/	94 vs. 92	/	22.7 vs. 17.5	/	/	/
ELOQUENT-3 [59]	eloPd vs. Pd	3 (2–8)	45	10.3 vs. 4.7	29.8 vs. 17.4	53 vs. 26	20 vs. 9	/	90 vs. 84	/	/	/	/	/
**CAR-T-based regimens**														
**Overall**									**Len-Refractory**					
**Study** **[Ref]**	**Regimens**	**Prior LOTs** **(m)**	**mFU** **(mos)**	**mPFS** **(mos)**	**mOS** **(mos)**	**ORR** **(%)**	**≥CR** **(%)**	**MRD-** **(%)**	**Len-Ref** **(%)**	**mPFS** **(mos)**	**mOS** **(mos)**	**ORR** **(%)**	**≥CR** **(%)**	**MRD-** **(%)**
CARTITUDE-4 [60,61]	cilta-cel vs. SOC	2 (1–3)	33.6	NR vs. 11.8 (30-mos PFS: 59.4% vs. 25.7%)	NR (30-mos OS: 76.4% vs. 63.8%)	85 vs. 67	77 vs. 24	62 vs. 19 (ITT) 89 vs. 38 (MRD evaluable pts)	All	/	/	/	/	/
KarMMa-3 [62,63]	ide-cel vs. SOC	6 (3–16)	30.9	13.8 vs. 4.4	41.4 vs. 37.9	71 vs. 42	44 vs. 6	35 vs. 2	73 vs. 79	/	/	/	/	/

Abbreviations: CR = complete response; DKd = daratumumab + carfilzomib + dexamethasone; DPd = daratumumab + pomalidomide + dexamethasone; DVd = daratumumab + bortezomib + dexamethasone; eloPd = elotuzumab + pomalidomide + dexamethasone; HR = hazard ratio; isaKd = isatuximab + carfilzomib + dexamethasone; isaPd = isatuximab + pomalidomide + dexamethasone; Kd = + carfilzomib + dexamethasone; Kd56 = + carfilzomib (56 mg/m^2^) + dexamethasone; len-ref = lenalidomide refractory patients; LOT = line(s) of therapy; m = median; mFU = median follow up; mOS = median overall survival; mPFS = median progression-free survival; MRD- = minimal residual disease negativity (10^−5^ sensitivity level); mos = months; NR = not reached; ORR = overall response rate; Pd = pomalidomide + dexamethasone; PVd = pomalidomide + bortezomib + dexamethasone; Ref = references; SOC = standard of care; SVd = selinexor + bortezomib + dexamethasone; Vd = bortezomib + dexamethasone.

**Table 2:** ADC-based triplet treatments for len-refractory MM currently under EMA evaluation: data on clinical efficacy.

			Overall						Len-Refractory					
Study [Ref]	Regimen	Prior LOTs (m)	mFU (mos)	mPFS (mos)	mOS (mos)	ORR (%)	≥CR (%)	MRD- (%)	Len-Ref (%)	mPFS (mos)	mOS (mos)	ORR (%)	≥CR (%)	MRD- (%)
DREAMM-7 [76,77,78,79]	BVd vs. DVd	≥1	39.4	36.6 vs. 13.4	NR in either group (36-mos OS: 74% vs. 60%)	83 vs. 71	34.6 vs. 17.1	39 vs. 17	33 vs. 35 (28 vs. 31 1 prior LOT)	25.0 vs. 8.6	/	84 vs. 61	35 vs. 11	42 vs. 13
DREAMM-8 [80,81]	BPd vs. PVd	≥1	21.8	NR vs. 12.7 (NR vs. 18.5 as 2nd LOT)	NR in either group (12-mos OS: 83% vs. 76%)	77 vs. 72	40 vs. 16	24 vs. 5 (CR) 32 vs. 5 (VGPR)	81 vs. 76	24.0 vs. 9.2 (NR vs. 13.1 as 2nd LOT)	NR in either group (12-mos OS: 82% vs. 72%)	75 vs. 68	38 vs. 14	23 vs. 5 (CR) 31 vs. 5 (VGPR)

Abbreviations: BPd = belantamab mafodotin + pomalidomide + dexamethasone; BVd = belantamab mafodotin + bortezomib + dexamethasone; CR = complete response; DVd = daratumumab + bortezomib + dexamethasone; len = lenalidomide; m = median; LOT = line(s) of therapy; mFU = median follow-up; mOS = median overall survival; mPFS = median progression-free survival; MRD- = minimal residual disease negativity (10^−5^ sensitivity level); mos = months; NR = not reached; ORR = overall response rate; PVd = pomalidomide + bortezomib + dexamethasone; Ref = references; VGPR = very good partial response.
