# Correction: Mancuso et al. Forcing Ahead: Second-Line Treatment Options for Lenalidomide-Refractory Multiple Myeloma. *Cancers* 2025, *17*, 1168

**DOI:** 10.3390/cancers17152555

**Published:** 2025-08-01

**Authors:** Katia Mancuso, Simona Barbato, Francesco Di Raimondo, Francesca Gay, Pellegrino Musto, Massimo Offidani, Maria Teresa Petrucci, Elena Zamagni, Renato Zambello, Michele Cavo

**Affiliations:** 1IRCCS Azienda Ospedaliero-Universitaria di Bologna, Istituto di Ematologia “Seràgnoli”, 40138 Bologna, Italy; katia.mancuso3@unibo.it (K.M.); simona.barbato3@unibo.it (S.B.); e.zamagni@unibo.it (E.Z.); 2Dipartimento di Scienze Mediche e Chirurgiche, Università di Bologna, 40138 Bologna, Italy; 3Divisione di Ematologia, Azienda Ospedaliero-Universitaria Policlinico di Catania, Scuola di Specializzazione in Ematologia dell’università di Catania, 95125 Catania, Italy; diraimon@unict.it; 4Divisione di Ematologia 1, AOU Città Della Salute e Della Scienza di Torino, 10126 Torino, Italy; francesca.gay@unito.it; 5Dipartimento di Biotecnologie Molecolari e Scienze per la Salute, Università degli Studi di Torino, 10126 Torino, Italy; 6Unit of Hematology and Stem Cell Transplantation, Department of Precision and Regenerative Medicine and Ionian Area, “Aldo Moro” University School of Medicine, AOUC Policlinico, 70124 Bari, Italy; pellegrino.musto@uniba.it; 7Clinica di Ematologia, Unità di Trapianto di Cellule Staminali e Terapia Cellulare dell’AOU delle Marche, 60126 Ancona, Italy; massimo.offidani@ospedaliriuniti.marche.it; 8Ematologia, Azienda Ospedaliera Universitaria Policlinico Umberto I, 00161 Roma, Italy; petrucci@bce.uniroma1.it; 9Unità di Ematologia, Dipartimento di Medicina (DIMED), Università di Padova, 65100 Padova, Italy; r.zambello@unipd.it

## Affiliations

The authors have separated the affiliations originally listed in affiliation 4—this is so both affiliations can be organized from subordinate to superior.

## References

In the original publication [1], there was a mistake, not attributable to the authors, in the order of the bibliographical references. With this correction, the order of some references in the text and tables has been corrected and aligned with the list of references at the end of the manuscript. 

## Tables

In the original publication [1], the titles and contents of Tables 1 and 2 were not up-to-date with the final version of the manuscript, as requested by the Authors. The corrected tables “Table 1. Currently licensed treatment strategies in len-refractory MM: data on clinical efficacy” and “Table 2. ADC-based triplet treatments for len-refractory MM currently under EMA evaluation: data on clinical efficacy” appear below.

## Text Correction

There was text correction made to the following sentence in Section 2—‘In a study aimed at gaining insights into the different patterns of len resistance, a longer exposure (≥1 year) to len and longer interval (≥18 months) from last len dose to subsequent start of pomalidomide were associated with higher response rates and longer PFS and OS, highlighting the importance of optimal sequencing to face the challenge of IMiD refractoriness [5]’.

The subheading for Section 4.1 has been updated to ‘PI-Based Doublets and Triplets Incorporating Either an Anti-CD38 MoAb or an Inhibitor of Exportin 1’.

In Section 4.2 there has been a text correction to paragraph 4—‘The combinations of Pd with MoAbs targeting CD38 and SLAMF7, like isa or elo (i.e., isa-Pd and elo-Pd, respectively), may be considered in heavily pretreated patients’.

There has been a text correction to Section 5, paragraph 5—‘Notably, at the second interim analysis, mOS was not yet reached in both arms, and 36-month estimations of OS were 74% in the BVd group and 60% in the DVd group, suggesting an early and statistically significant benefit with BVd (the HR was 0.58 [95% CI, 0.43–0.79]) [79]’.

The authors state that the scientific conclusions are unaffected. This correction was approved by the Academic Editor. The original publication has also been updated.

## Figures and Tables

**Table 1 cancers-17-02555-t001:** Currently licensed treatment strategies in len-refractory MM: data on clinical efficacy.

PI-Based Regimens														
Overall									Len-Refractory					
**Study** **[Ref]**	**Regimens**	**Prior LOTs** **(m)**	**mFU** **(mos)**	**mPFS** **(mos)**	**mOS** **(mos)**	**ORR** **(%)**	**≥CR** **(%)**	**MRD-** **(%)**	**Len-Ref** **(%)**	**mPFS** **(mos)**	**mOS** **(mos)**	**ORR** **(%)**	**≥CR** **(%)**	**MRD-** **(%)**
ENDEAVOR [30–32]	Kd56 vs. Vd	2 (1–3)	44.3	18.7 vs. 9.4	47.8 vs. 38.8 (51.3 vs. 43.7 as 2nd LOT)	77 vs. 63	13 vs. 6	/	25	8.6 vs. 6.6	29.2 vs. 21.4	/	15.5 (Kd56)	/
CASTOR [33–35]	DVd vs. Vd	2 (1–9)	72.6	16.7 vs. 7.1 (27.0 vs. 7.9 as 2nd LOT)	49.6 vs. 38.5	84 vs. 63	29 vs. 10	14 vs. 2	24	7.8 vs. 4.9	/	/	/	/
CANDOR [36–38]	DKd vs. Kd	2 (1–5)	50.6	28.4 vs. 15.2	50.8 vs. 43.6	84 vs. 73	22 vs. 8	28 vs. 9	32 vs. 36	28.1 vs. 11.1	NR vs. 38.2	79.8 (DKd)	/	/
IKEMA [39–44]	isaKd vs. Kd	2 (1–4)	56.6	35.7 vs. 19.2	NR vs. 50.6	87 vs. 84	44 vs. 29	34 vs. 15	32 vs. 34	HR 0.60 in favor of isaKd	/	/	39 vs. 12	25 vs. 10
BOSTON [45,46]	SVd vs. Vd	2 (1–3)	28	13.2 vs. 9.5 (21.0 vs. 10.7 as 2nd LOT)	36.7 vs. 32.8 (NR vs. 32.8 as 2nd LOT)	76 vs. 62	17 vs. 11	/	37 vs. 39	10.2 vs. 7.1	26.7 vs. 18.6	67.9 vs. 47.2	/	/
**IMiD** **-based regimens**														
**Overall**									**Len-Refractory**					
**Study** **[Ref]**	**Regimens**	**Prior LOTs** **(m)**	**mFU** **(mos)**	**mPFS** **(mos)**	**mOS** **(mos)**	**ORR** **(%)**	**≥CR** **(%)**	**MRD-** **(%)**	**Len-ref** **(%)**	**mPFS** **(mos)**	**mOS** **(mos)**	**ORR** **(%)**	**≥CR** **(%)**	**MRD-** **(%)**
OPTIMISMM [47–49]	PVd vs. Vd	2 (1–3)	64.5	11.7 vs. 6.9 (20.73 vs. 11.63 as 2nd LOT)	35.6 vs. 31.6	82 vs. 50	13 vs. 3	/	71 vs. 69	17.8 vs. 9.5 (2nd LOT)	29.8 vs. 24.2	85.9 vs. 50.8	/	/
APOLLO [50–52]	DPd vs. Pd	2 (1–5)	39.6	12.4 vs. 6.9	34.4 vs. 23.7	69 vs. 46	25 vs. 4	9 vs. 2	63 vs. 73	9.9 vs. 6.5	/	/	/	/
MM-014 [53,54]	DPd	2 (1–2)	41.9	23.7	56.7	78.6	26.8	/	76	23.0	53.6	77.6	22.4	/
ICARIA [55–58]	isaPd vs. Pd	3 (2–4)	52.4	11.5 vs. 6.5	24.6 vs. 17.7	60 vs. 35	9 vs. 2	/	94 vs. 92	/	22.7 vs. 17.5	/	/	/
ELOQUENT-3 [59]	eloPd vs. Pd	3 (2–8)	45	10.3 vs. 4.7	29.8 vs. 17.4	53 vs. 26	20 vs. 9	/	90 vs. 84	/	/	/	/	/
**CAR-T-based regimens**														
**Overall**									**Len-Refractory**					
**Study** **[Ref]**	**Regimens**	**Prior LOTs** **(m)**	**mFU** **(mos)**	**mPFS** **(mos)**	**mOS** **(mos)**	**ORR** **(%)**	**≥CR** **(%)**	**MRD-** **(%)**	**Len-Ref** **(%)**	**mPFS** **(mos)**	**mOS** **(mos)**	**ORR** **(%)**	**≥CR** **(%)**	**MRD-** **(%)**
CARTITUDE-4 [60,61]	cilta-cel vs. SOC	2 (1–3)	33.6	NR vs. 11.8 (30-mos PFS: 59.4% vs. 25.7%)	NR (30-mos OS: 76.4% vs. 63.8%)	85 vs. 67	77 vs. 24	62 vs. 19 (ITT) 89 vs. 38 (MRD evaluable pts)	All	/	/	/	/	/
KarMMa-3 [62,63]	ide-cel vs. SOC	6 (3–16)	30.9	13.8 vs. 4.4	41.4 vs. 37.9	71 vs. 42	44 vs. 6	35 vs. 2	73 vs. 79	/	/	/	/	/

Abbreviations: CR = complete response; DKd = daratumumab + carfilzomib + dexamethasone; DPd = daratumumab + pomalidomide + dexamethasone; DVd = daratumumab + bortezomib + dexamethasone; eloPd = elotuzumab + pomalidomide + dexamethasone; HR = hazard ratio; isaKd = isatuximab + carfilzomib + dexamethasone; isaPd = isatuximab + pomalidomide + dexamethasone; Kd = + carfilzomib + dexamethasone; Kd56 = + carfilzomib (56 mg/m^2^) + dexamethasone; len-ref = lenalidomide refractory patients; LOT = line(s) of therapy; m = median; mFU = median follow up; mOS = median overall survival; mPFS = median progression-free survival; MRD- = minimal residual disease negativity (10^−5^ sensitivity level); mos = months; NR = not reached; ORR = overall response rate; Pd = pomalidomide + dexamethasone; PVd = pomalidomide + bortezomib + dexamethasone; Ref = references; SOC = standard of care; SVd = selinexor + bortezomib + dexamethasone; Vd = bortezomib + dexamethasone.

**Table 2 cancers-17-02555-t002:** ADC-based triplet treatments for len-refractory MM currently under EMA evaluation: data on clinical efficacy.

			Overall						Len-Refractory					
Study [Ref]	Regimen	Prior LOTs (m)	mFU (mos)	mPFS (mos)	mOS (mos)	ORR (%)	≥CR (%)	MRD- (%)	Len-Ref (%)	mPFS (mos)	mOS (mos)	ORR (%)	≥CR (%)	MRD- (%)
DREAMM-7 [76–79]	BVd vs. DVd	≥1	39.4	36.6 vs. 13.4	NR in either group (36-mos OS: 74% vs. 60%)	83 vs. 71	34.6 vs. 17.1	39 vs. 17	33 vs. 35 (28 vs. 31 1 prior LOT)	25.0 vs. 8.6	/	84 vs. 61	35 vs. 11	42 vs. 13
DREAMM-8 [80,81]	BPd vs. PVd	≥1	21.8	NR vs. 12.7 (NR vs. 18.5 as 2nd LOT)	NR in either group (12-mos OS: 83% vs. 76%)	77 vs. 72	40 vs. 16	24 vs. 5 (CR) 32 vs. 5 (VGPR)	81 vs. 76	24.0 vs. 9.2 (NR vs. 13.1 as 2nd LOT)	NR in either group (12-mos OS: 82% vs. 72%)	75 vs. 68	38 vs. 14	23 vs. 5 (CR) 31 vs. 5 (VGPR)

Abbreviations: BPd = belantamab mafodotin + pomalidomide + dexamethasone; BVd = belantamab mafodotin + bortezomib + dexamethasone; CR = complete response; DVd = daratumumab + bortezomib + dexamethasone; len = lenalidomide; m = median; LOT = line(s) of therapy; mFU = median follow-up; mOS = median overall survival; mPFS = median progression-free survival; MRD- = minimal residual disease negativity (10^−5^ sensitivity level); mos = months; NR = not reached; ORR = overall response rate; PVd = pomalidomide + bortezomib + dexamethasone; Ref = references; VGPR = very good partial response.
